# Supplementary material for: PrPres in placental tissue following experimental transmission of atypical scrapie in ARR/ARR sheep is not infectious by Tg338 mouse bioassay
Source: PLoS One. 2022 Jan 21;17(1):e0262766. doi: 10.1371/journal.pone.0262766 (PMC8782414; doi:10.1371/journal.pone.0262766)
Supplement: S1 Raw images — (PDF) [file pone.0262766.s001.pdf]

Internal filing information redacted

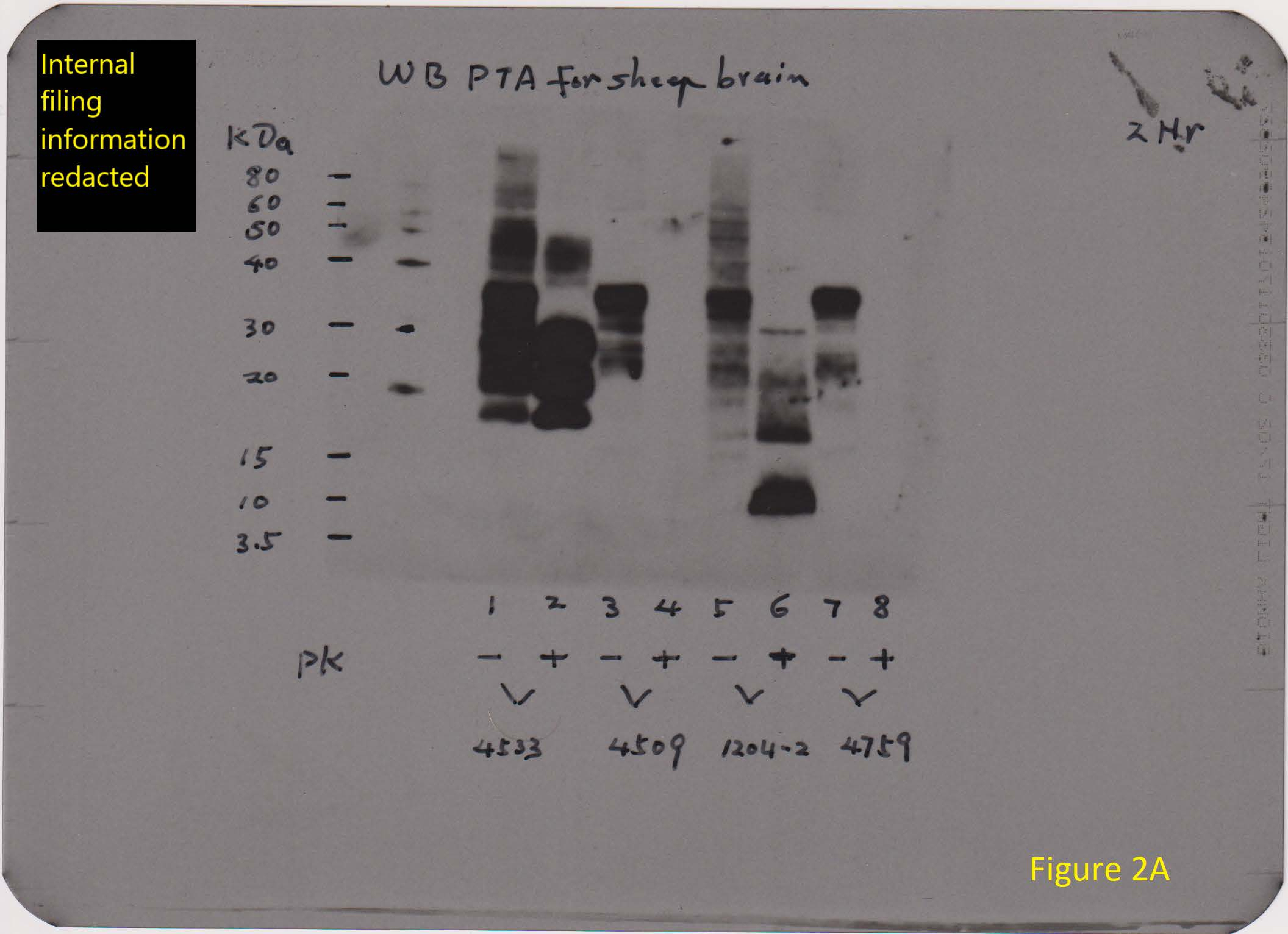

Figure 2A

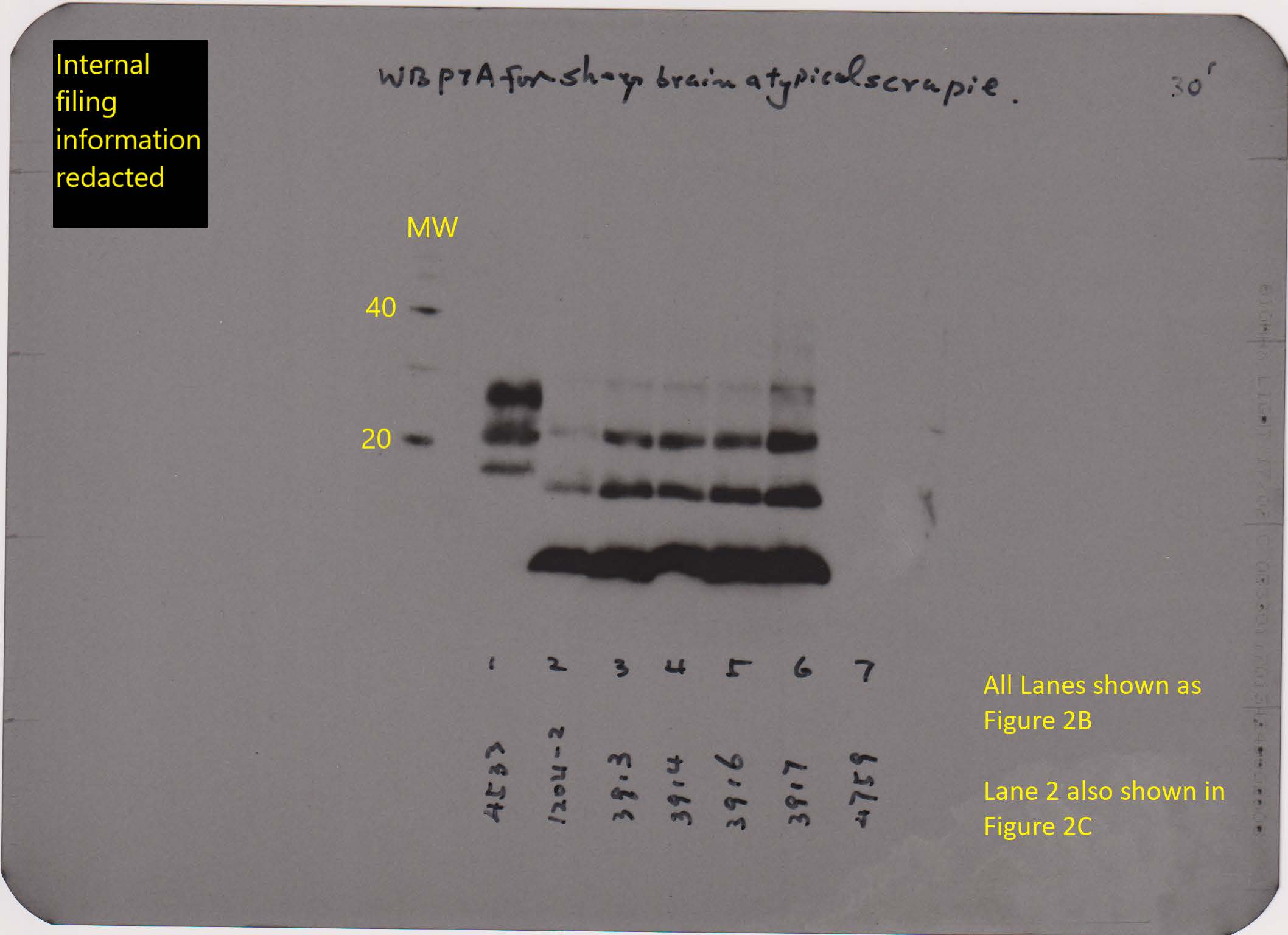
Internal filing information redacted
WB PTA for sheep brain atypical scrapie.
30'
MW
40
20
1 2 3 4 5 6 7
All Lanes shown as Figure 2B
Lane 2 also shown in Figure 2C

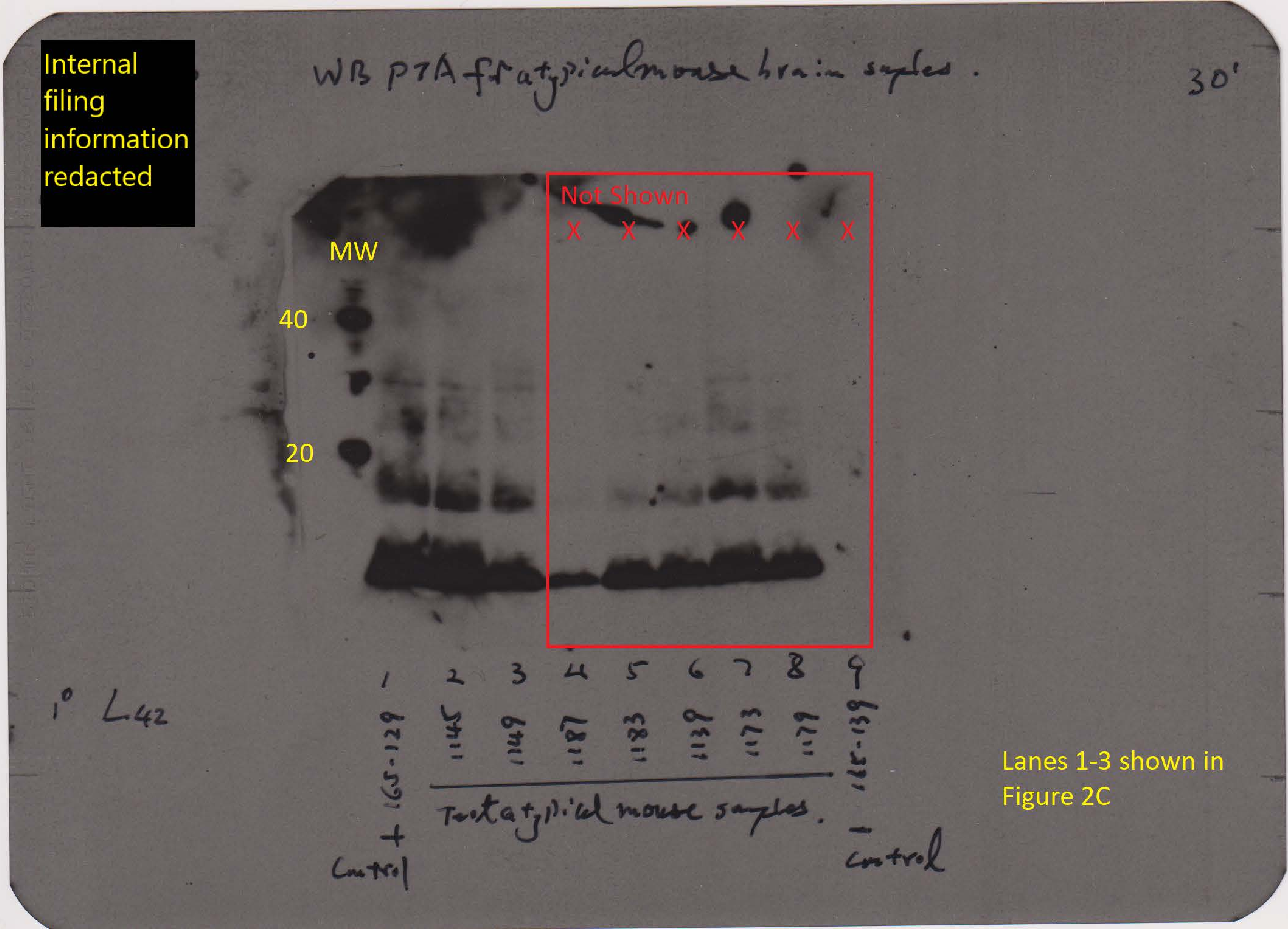
Internal filing information redacted
WB P7A for atypical mouse brain samples.
30'
Not Shown
X X X X X X
MW
40
20
1 2 3 4 5 6 7 8 9
1° L42
Test atypical mouse samples.
+ Control
- Control
Lanes 1-3 shown in Figure 2C

Internal filing information redacted

atypical shp brn to ms brn (PTA)
1 min (PTA)

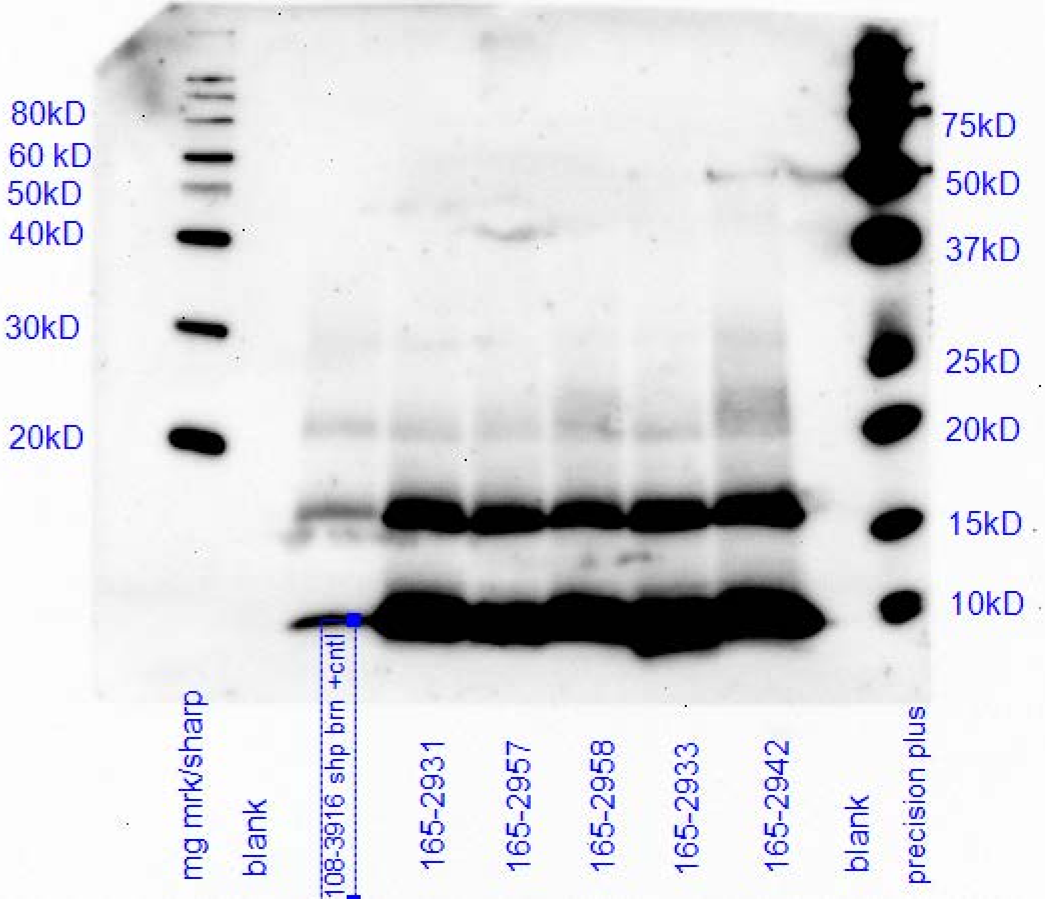

Figure 2D

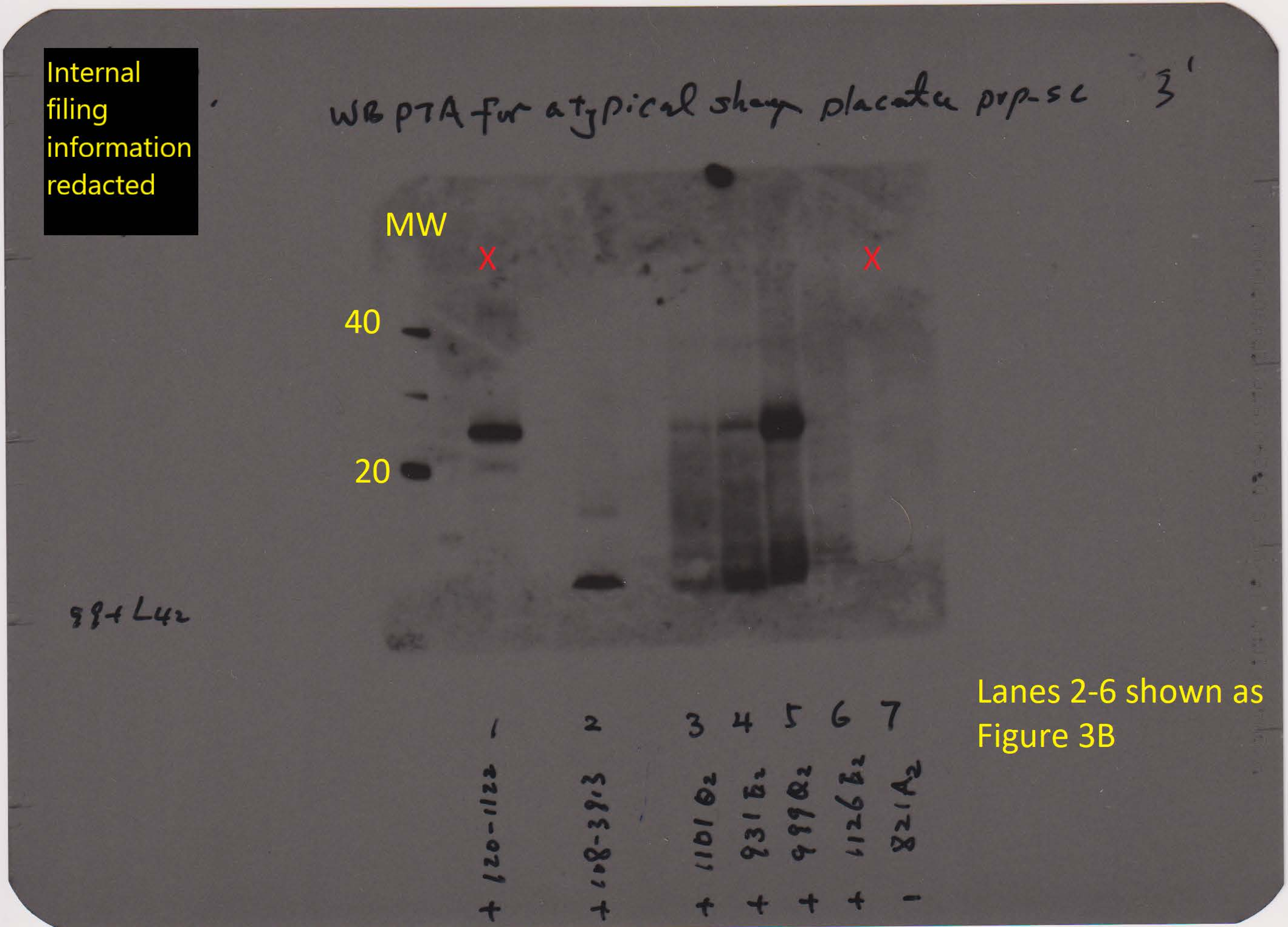
Internal filing information redacted
WB PTA for atypical sheep placenta prp-sc 3'
MW
X
X
40
20
1 2 3 4 5 6 7
Lanes 2-6 shown as Figure 3B

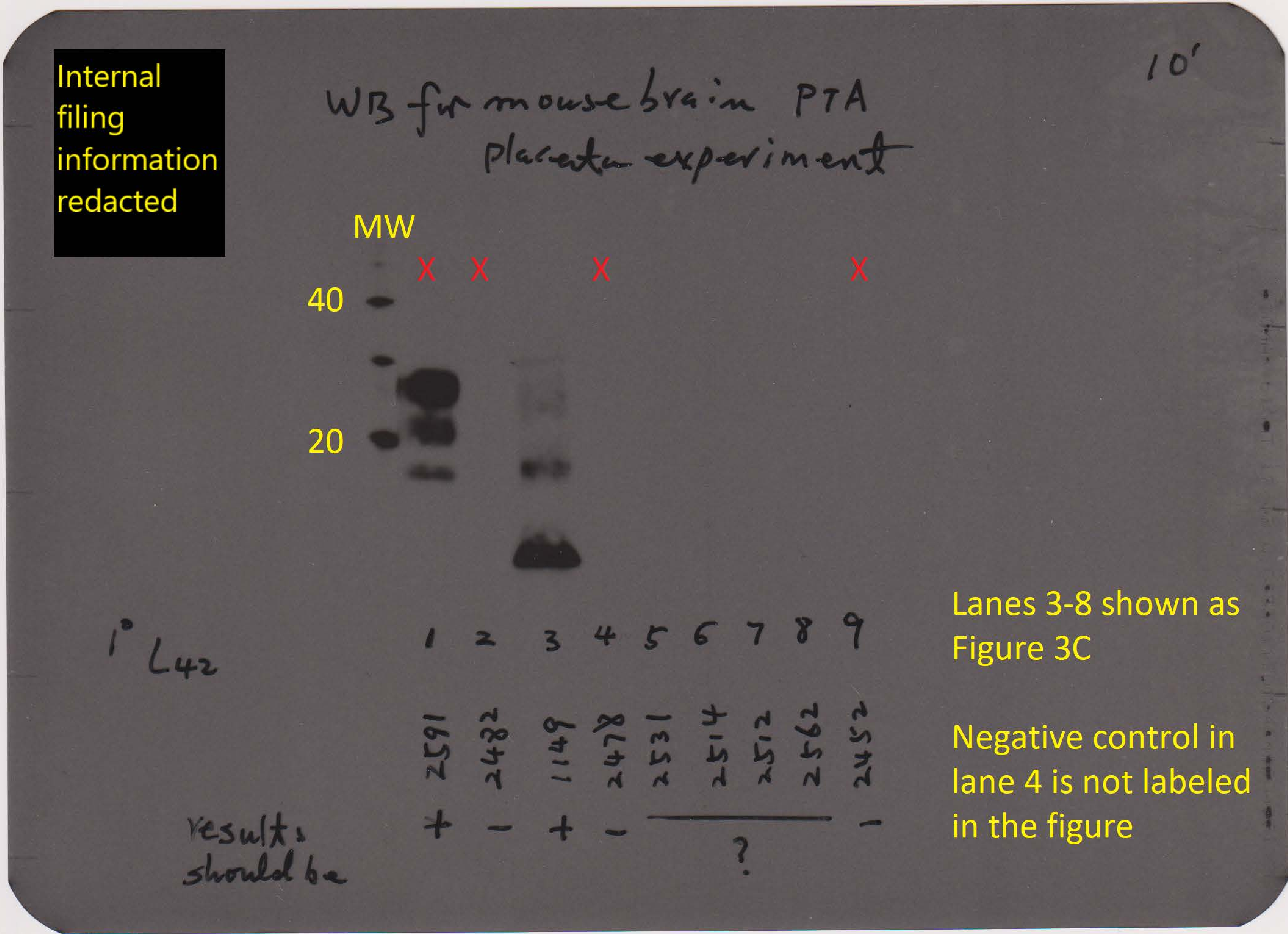
Internal filing information redacted
10'
WB for mouse brain PTA placenta experiment
MW
X X X X
40
20
1° L42
1 2 3 4 5 6 7 8 9
2591 2482 1149 2478 2531 2514 2512 2562 2452
results should be
+ − + − ? −
Lanes 3-8 shown as Figure 3C
Negative control in lane 4 is not labeled in the figure
