# Supplementary material for: PrPres in placental tissue following experimental transmission of atypical scrapie in ARR/ARR sheep is not infectious by Tg338 mouse bioassay
Source: PLoS One. 2022 Jan 21;17(1):e0262766. doi: 10.1371/journal.pone.0262766 (PMC8782414; doi:10.1371/journal.pone.0262766)
Supplement: Attachment — Submitted filename: Response to Reviewers.docx [file pone.0262766.s002.docx]

Please find editor and reviewer comments and our responses (in blue) below:

**Editor 1.** Please ensure that your manuscript meets PLOS ONE's style requirements, including those for file naming. The PLOS ONE style templates can be found at

https://journals.plos.org/plosone/s/file?id=wjVg/PLOSOne_formatting_sample_main_body.pdf and

https://journals.plos.org/plosone/s/file?id=ba62/PLOSOne_formatting_sample_title_authors_affiliations.pdf

**We have reviewed the style templates and believe our submitted manuscript conforms to all requirements.**

**Editor 2.** PLOS ONE has specific criteria regarding the reporting of animal research (https://journals.plos.org/plosone/s/submission-guidelines#loc-animal-research). Specifically, these guidelines require that details regarding care, monitoring, and method of sacrifice are clearly stated. As part of your revision, please complete and submit a copy of the ARRIVE Guidelines checklist, a document that aims to improve experimental reporting and reproducibility of animal studies for purposes of post-publication data analysis and reproducibility: https://arriveguidelines.org/sites/arrive/files/Author%20Checklist%20-%20Full.pdf. Please include your completed checklist as a Supporting Information file. Note that if your paper is accepted for publication, this checklist will be published as part of your article.

**Our completed ARRIVE Guidelines checklist has been submitted as a Supporting Information file.**

**Editor 3.** Thank you for stating the following in the Acknowledgments Section of your manuscript:

[The authors wish to thank the following people for providing excellent technical support to this project: Tom Truscott, Lori Fuller, Laetisha O’Rourke, Jian Zhang. The authors greatly appreciate the consistently excellent animal care provided by numerous staff members over the extended years of this project. Special appreciation is given to Dr. Katherine O’Rourke who participated in the initial design and management of this study. This work was funded by the USDA-Agricultural Research Service under CRIS 2090-32000-035-000-D. Mention of trade names or commercial products in this article is solely for the purpose of providing specific information and does not imply recommendation or endorsement by the US Department of Agriculture.]

We note that you have provided funding information that is not currently declared in your Funding Statement. However, funding information should not appear in the Acknowledgments section or other areas of your manuscript. We will only publish funding information present in the Funding Statement section of the online submission form.

Please remove any funding-related text from the manuscript and let us know how you would like to update your Funding Statement. Currently, your Funding Statement reads as follows:

[DAS, 2090-32000-035-000-D, US Department of Agriculture, Agricultural Research Service, https://www.ars.usda.gov/research/project/?accnNo=431730. The funders had no role in study design, data collection and analysis, decision to publish, or preparation of the manuscript.]

Please include your amended statements within your cover letter; we will change the online submission form on your behalf.

**Funding information had been removed from the acknowledgements; our Funding Statement is accurate as submitted and amendment is not necessary.**

**Editor 4.** PLOS ONE now requires that authors provide the original uncropped and unadjusted images underlying all blot or gel results reported in a submission’s figures or Supporting Information files. This policy and the journal’s other requirements for blot/gel reporting and figure preparation are described in detail at https://journals.plos.org/plosone/s/figures#loc-blot-and-gel-reporting-requirements and https://journals.plos.org/plosone/s/figures#loc-preparing-figures-from-image-files. When you submit your revised manuscript, please ensure that your figures adhere fully to these guidelines and provide the original underlying images for all blot or gel data reported in your submission. See the following link for instructions on providing the original image data: https://journals.plos.org/plosone/s/figures#loc-original-images-for-blots-and-gels.

In your cover letter, please note whether your blot/gel image data are in Supporting Information or posted at a public data repository, provide the repository URL if relevant, and provide specific details as to which raw blot/gel images, if any, are not available. Email us at plosone@plos.org if you have any questions.

**Uncropped and unadjusted images have been provided in Supporting Information as “S1_raw_images”.**

**Editor 5.** We note that you have included the phrase “data not shown” in your manuscript. Unfortunately, this does not meet our data sharing requirements. PLOS does not permit references to inaccessible data. We require that authors provide all relevant data within the paper, Supporting Information files, or in an acceptable, public repository. Please add a citation to support this phrase or upload the data that corresponds with these findings to a stable repository (such as Figshare or Dryad) and provide and URLs, DOIs, or accession numbers that may be used to access these data. Or, if the data are not a core part of the research being presented in your study, we ask that you remove the phrase that refers to these data.

**The phrase “data not shown” has been removed from line 508. The statement in which “data not shown” was included has been left in the manuscript as it is a statement explaining a choice in methodology, rather than presenting any data from which conclusions were drawn.**

**Editor 6.** Please review your reference list to ensure that it is complete and correct. If you have cited papers that have been retracted, please include the rationale for doing so in the manuscript text, or remove these references and replace them with relevant current references. Any changes to the reference list should be mentioned in the rebuttal letter that accompanies your revised manuscript. If you need to cite a retracted article, indicate the article’s retracted status in the References list and also include a citation and full reference for the retraction notice.

**A reference error was found and corrected on line 397.**

**Reviewer 1-1.** Line 91-99 and Lines 431-435: Are the sheep infection studies/tissues used for this manuscript harvested from sheep inoculated in the original publication (Reference 7; Loiacono et. al.)? If so, please mention in Materials and Methods.

**We have attempted to clarify the language in line 93. This information can be found in lines 447-452 of the Materials and Methods section under the heading “Original Nor98-like scrapie case material”.**

**Reviewer 1-2.** Line 121 and line 127: Maintain consistency in reporting Tg or tg.

**We have changed all instances to “Tg”.**

**Reviewer 1-3.** Lines 392-394: Do atypical scrapie prions circulate in the periphery via blood transport? As the authors point out there is presence of infectivity in lymphoid tissue harvested from atypical scrapie cases. This reviewer agrees that ‘they are relatively unlikely to be a source of prion shedding’, yet, as infectivity is present in lymphoid tissue, it may spillover into the peripheral blood supply to organs associated with prion shedding in saliva, urine, feces, etc. This could result in the shedding of PK resistant prions in bodily fluids/excretions to the environment. Please comment within the discussion that this may be the case.

**A discussion of this has been added beginning at line 399.**

**Reviewer 1-4.** Lines 401-404 and 408-409: The difference may also be due to the route of inoculation, IC, vs a more natural route of inoculation/infection. Please indicate within the discussion the impact the route of inoculation may have on your results.

**The differences discussed in this section are comparing banding patterns from brain and cotyledon tissues from the same animals, all of which were inoculated IC. Since all animals being compared were inoculated by the same route, we cannot attribute the observed differences to inoculation route.**

**Reviewer 2-1.** My only suggestions are minor and consist of requests to add symbolic annotations (arrows, arrowheads, etc...) to figures 1D and 1E to point out the PrPsc accumulations in 2 regions (1D) and plaques and glial cells (1E).

**The requested annotations have been added to Figures 1D and 1E and listed in the figure legend at lines 114-118.**
